# Vector Competence of *Aedes caspius* and *Ae. albopictus* Mosquitoes for Zika Virus, Spain

**DOI:** 10.3201/eid2502.171123

**Published:** 2019-02

**Authors:** Rafael Gutiérrez-López, Sean M. Bialosuknia, Alexander T. Ciota, Tomás Montalvo, Josue Martínez-de la Puente, Laura Gangoso, Jordi Figuerola, Laura D. Kramer

**Affiliations:** Estación Biológica de Doñana, Seville, Spain (R. Gutiérrez-López, J. Martínez-de la Puente, L. Gangoso, J. Figuerola);; New York State Department of Health, Slingerlands, New York, USA (S.M. Bialosuknia, A.T. Ciota, L.D. Kramer);; Agència de Salut Pública de Barcelona, Barcelona, Spain (T. Montalvo);; Centro de Investigación Biomédica en Red de Epidemiología y Salud Pública (CIBERESP), Barcelona (T. Montalvo, J. Martínez-de la Puente, J. Figuerola)

**Keywords:** vector-borne infections, vertical transmission, Zika virus, Spain, viruses, *Aedes caspius*, *Aedes albopictus*, mosquitoes

## Abstract

We assessed the vector competence of *Aedes caspius* and *Aedes albopictus* mosquitoes in Spain for the transmission of Zika virus. Whereas *Ae. albopictus* mosquitoes were a competent vector, *Ae. caspius* mosquitoes were unable to transmit Zika virus. We also identified high levels of vertical transmission of Zika virus in *Ae. albopictus* mosquitoes.

Zika virus is an emerging arbovirus of the family *Flaviviridae* primarily transmitted by *Aedes aegypti* mosquitoes, but other *Aedes* species mosquitoes could be competent vectors (*1*). *Ae. aegypti* mosquitoes are absent from most countries in Europe (*2*), and the invasive *Ae. albopictus* mosquito and other native species could create novel epidemiologic scenarios for Zika virus. Indeed, *Ae. albopictus* mosquito populations from Europe are competent vectors for Zika virus (*3**,**4*). However, the vector competence for transmission of Zika virus of most mosquito species of Europe is currently unknown and may vary across virus strains and mosquito populations (*5*).

Although no autochthonous vectorborne Zika virus transmission has been reported in Spain, >316 imported cases of Zika virus have been confirmed (*6*). The confirmed cases, together with the presence of both the *Ae. albopictus* mosquito (*7*) and the native *Ae. caspius* mosquito (*8**,**9*) (a potential vector of chikungunya virus [*10*] and Rift Valley fever virus [*11*]), indicate a risk for Zika virus transmission in Spain. Accurately quantifying this risk requires evaluating the competence of these mosquito species for Zika virus.

We determined vector competence at different days postinfection (dpi) by exposing F0 generation of *Ae. caspius* mosquitoes (collected as larvae in Huelva, Spain, because we were unable to rear it under laboratory conditions) and F2 generation of *Ae. albopictus* mosquitoes (collected as eggs in Barcelona, Spain) to Zika virus through infectious blood meals. We used F8 generation of colonized populations of *Ae. aegypti* mosquitoes (collected in Poza Rica, Mexico) as a control population and Zika virus strains CAM (2010 Cambodia; GenBank accession no. JN860885) and PR (2015 Puerto Rico; GenBank accession no. KU501215), passaged 4 times on Vero cells and 2 times on C6/36 cells. We propagated on C6/36 cells for 4 days, and freshly harvested supernatant was mixed 1:1 with sheep blood (Colorado Serum Company, http://www.thepeakofquality.com) and 2.5% sucrose (*5*).

We offered to 4- to 7-day-old *Ae. albopictus* and *Ae. aegypti* female mosquitoes infectious blood meals containing either the CAM or PR strain at a final concentration of 7.6 log_10_ PFU/mL. Infection rates were determined by screening mosquitoes’ bodies, dissemination rates by screening legs, and transmission rates by screening saliva, at 3 different time points (7, 14 and 21 dpi) using Zika-specific quantitative reverse transcription PCR including negative controls in each reaction (*12*) (Table 1; Appendix). We calculated Zika titers from standard curves on the basis of infectious particle standards created from matched virus stocks (*5*).

**Table 1:** Infection, dissemination, and transmission rates of mosquitoes experimentally infected with 2 Zika virus strains, Spain

Days postinfection	Mosquito species	Zika virus strain*	Blood meal titers, log_10_ PFU/mL	% Infected (total no.)	% Infected disseminating	% Infected transmitting
7	*Aedes aegypti*	CAM	7.6	24.2 (33)	75	12.5
		PR	7.6	61.8 (34)	38.1	0
	*Ae. albopictus*	CAM	7.6	90.5 (21)	42	10.5
		PR	7.6	97.0 (33)	31.3	0
	*Ae. caspius*	PR	7.7	21.4 (14)	0	0
14	*Ae. aegypti*	CAM	7.6	22.6 (31)	71.4	14.3
		PR	7.6	45 (40)	77.8	16.7
	*Ae. albopictus*	CAM	7.6	81.5 (27)	81.8	9.1
		PR	7.6	93.3 (30)	67.9	0
	*Ae. caspius*	PR	8.7	40 (25)	0	0
21	*Ae. aegypti*	CAM	7.6	35.7 (28)	100	40
		PR	7.6	56.3 (32)	88.9	38.9
	*Ae. albopictus*	CAM	7.6	94.4 (18)	82.4	23.6
		PR	7.6	96.2 (26)	96	36
	*Ae. caspius*	PR	7.6	18.5 (27)	0	0

*CAM, Zika virus 2010 Cambodia strain; PR, Zika virus 2015 Puerto Rico strain.

We further exposed 4- to 10-day-old *Ae. caspius* female mosquitoes to the PR strain as described. We conducted 3 independent trials using different Zika virus concentrations at different time points (7, 14, or 21 dpi) for each trial (Table 1; Appendix).

To determine the ability of *Ae. albopictus* mosquitoes to vertically transmit Zika virus, 4- to 7-day-old females were infected with Zika PR as described, and noninfectious blood meals were offered weekly after the first oviposition. We collected eggs laid in the second oviposition and hatched them for subsequent testing. We grouped second instar larvae in pools of 5 individuals and tested them for Zika virus (*13*). We estimated vertical transmission rate, measured as filial infection rate using the maximum-likelihood method (PoolInfRate version 4.0, https://www.cdc.gov/westnile/resourcepages/mosqSurvSoft.html) (*13*).

We performed generalized linear models with binomial error distribution and logit link function to assess the effect of mosquito species, virus strains, and dpi on the infection, dissemination, and transmission rates. We also considered the interactions between virus strain and dpi and between virus strain and mosquito species. We determined differences in mean viral titers between mosquito species, virus strains, and dpi in mosquito body, legs, and saliva using Kruskal-Wallis tests. Analyses were run in JMP version 9 (SAS Institute, http://www.jmp.com).

Infection rate was higher in *Ae. albopictus* than in *Ae. aegypti* mosquitoes, and Zika PR had a higher infection rate than Zika CAM. Dissemination rate increased with time (dpi) but was similar between mosquito species and Zika strains. Transmission rate also increased with time, and mosquitoes infected with Zika CAM showed a higher transmission rate than those infected with Zika PR. Transmission rate did not differ between *Ae. albopictus* and *Ae. aegypti* mosquitoes (Table 1,2). Mean viral titers in bodies differed between mosquito species and Zika strains, with higher titers in *Ae. albopictus* compared with *Ae. aegypti* mosquitoes (χ^2^ = 5.09, df = 1; p<0.02) and higher titers for Zika PR compared with Zika CAM (χ^2^ = 6.92, df = 1; p<0.009). Mean viral titers in legs were similar for both Zika strains (χ^2^ = 0.95, df = 1; p = 0.33), but were higher in *Ae. aegypti* relative to *Ae. albopictus* mosquitoes (χ^2^ = 9.53, df = 1; p<0.002). Mean viral titers did not differ in saliva secretions between mosquito species (χ^2^ = 1.7, df = 1; p = 0.19) or Zika strains (χ^2^ = 1.02, df = 1; p = 0.31). We detected Zika virus infection in *Ae. caspius* mosquitoes at 7, 14, and 21 dpi, but detected no virus dissemination or transmission at any point (Table 1). Five larval pools of *Ae. albopictus* mosquitoes (29.4%; N = 17) were positive for Zika virus, with a filial infection rate of 72.2 (95% CI 27.6–156.1) and mean viral load of 2.5 log_10_ PFU/mL. This value equates to a ratio of 1:14.

**Table 2:** Results of generalized linear models analyzing the variation in infection, dissemination, and transmission rates of Zika virus*

Variable	Infection rate				Dissemination rate				Transmission rate		
	χ^2^	df	p value		χ^2^	df	p value		χ^2^	df	p value
Mosquito species	110.95	1	**<0.001**		2.08	1	0.15		2.37	1	0.12
Zika virus strain	10.43	1	**0.001**		1.28	1	0.26		4.91	1	**0.03**
dpi	0.15	1	0.70		39.61	1	**<0.001**		26.77	1	**<0.001**
Zika virus strain • dpi	1.17	1	0.28		1.34	1	0.25		6.70	1	**0.01**
Mosquito species • Zika virus strain	0.01	1	0.90		0.76	1	0.39		0.01	1	0.94

*Bold indicates significant effect; • indicates interaction between variables;. dpi, days postinfection.

Our results suggest *Ae. albopictus* mosquitoes in Spain are competent vectors of Zika virus at levels similar to *Ae. aegypti* mosquitoes. We detected Zika CAM in saliva earlier than Zika PR, which suggests that genetically variable strains may have different transmission potential (*5*). Although a similar transmission rate was found in *Ae. albopictus* mosquitoes from Spain and Italy (*3*), lower rates were measured in populations in France (*4*). In addition, *Ae. albopictus* mosquitoes from Spain could transmit Zika virus at 7 dpi, 4 days earlier than mosquitoes in Italy (*4*). These discrepancies may be explained by variation in vector competence between mosquito populations and virus strains (*5*). Although Zika virus can infect *Ae. caspius* mosquitoes, it is unable to escape the midgut and be effectively transmitted (*14*).

Zika virus is vertically transmitted by the population of *Ae. albopictus* mosquitoes in Spain at substantially higher rates than found in *Ae. albopictus* mosquitoes from New York and Italy (*4*,*13*) and for other flaviviruses (*15*). These results suggest that the ability of Zika virus to be transmitted vertically is highly population dependent and could contribute to maintenance of the virus in *Ae. albopictus* mosquitoes in Spain.

Our results confirm that populations of *Ae. albopictus* mosquitoes increase the risk for Zika virus transmission in Spain. The high number of imported Zika virus cases and the rapid spread of *Ae. albopictus* mosquitoes contribute to the risk for autochthonous transmission of Zika virus. The risk for transmission by *Ae. caspius* mosquitoes, however, may be considered extremely low.

Appendix Additional information about vector competence of *Aedes caspius* and *Ae. albopictus* mosquitoes for Zika virus, Spain.
