# Supplementary material for: Vector Competence of Aedes caspius and Ae. albopictus Mosquitoes for Zika Virus, Spain
Source: Emerg Infect Dis. 2019 Feb;25(2):346–8. doi: 10.3201/eid2502.171123 (PMC6346449; doi:10.3201/eid2502.171123)
Supplement: Appendix — Additional information about vector competence of Aedes caspius and Ae. albopictus mosquitoes for Zika virus, Spain. [file 17-1123-Techapp-s1.pdf]

Article DOI: https://doi.org/10.3201/eid2502.171123

Vector Competence of *Aedes caspius* and *Ae. albopictus* Mosquitoes for Zika Virus, Spain

Appendix

Mosquito Collection and Rearing

More than 2,000 eggs of *Aedes albopictus* mosquitoes were originally collected in clapboards in Barcelona (northeastern Spain) in summer 2016; they were shipped to the New York State Department of Health (NYSDOH) Arbovirus Laboratory (Albany, NY, USA) and subsequently colonized. *Aedes aegypti* mosquitoes (provided by Gregory Ebel, Colorado State University) were originally collected in Poza Rica, Mexico, and subsequently colonized in the NYSDOH Arbovirus Laboratory.

Additionally, 2 shipments were sent to the NYSDOH Arbovirus Laboratory, each with ≈1,000 larvae of *Aedes caspius* mosquitoes collected in marshlands of the Huelva province (southern Spain) in summer and autumn 2016.

All mosquitoes were fed *ad libitum* with a 10% sugar solution and maintained under standard insectary conditions.

Oral Infection and Processing of Mosquitoes

All female mosquitoes were starved for 24 hours and subsequently fed with an infectious blood meal warmed to 37°C using a Hemotek feeding system (Discovery Workshops, http://hemotek.co.uk/).

F0 *Ae. caspius* female mosquitoes were infected with Zika virus 2015 Puerto Rico (PR) in 3 independent trials. Ninety-two *Ae. caspius* mosquitoes were exposed to ZIKV (blood meal titer = 8.7 $\log_{10}$ PFU/mL) in the first infection trial. Of those, 31 were completely engorged and the competence for the transmission of Zika virus was determined at 14 days postinfection (dpi).

In the second trial, 61 *Ae. caspius* mosquitoes were exposed to Zika virus (blood meal titer = 7.7 Log_{10} PFU/mL) and 14 of them were completely engorged. These mosquitoes were used to determine competence of Zika virus at 7 dpi. In the third trial, 65 *Ae. caspius* mosquitoes were exposed to ZIKV (blood meal titer = 7.6 Log_{10} PFU/mL) and 32 of them were completely engorged. These mosquitoes were used to determine competence of Zika virus at 21 dpi.

Approximately 2,500 F2 *Ae. albopictus* and 2,500 F8 *Ae. aegypti* mosquitoes were exposed to Zika virus PR (blood titer = 7.6 log_{10} PFU/mL) and Zika virus CAM (blood titer = 7.6 log_{10} PFU/mL). Approximately 150 mosquitoes of each species were engorged with infected blood. Mosquitoes were housed in independent containers and competence for Zika virus was determined at 7, 14, and 21 dpi.

RT-qPCR Protocol for Virus Detection

We used the protocol detailed by Lanciotti et al. (*1*). In brief, the protocol consisted in a RT-qPCR reaction containing 5 µL of extracted RNA as template, 13 µL 2x Master Mix Quanta QScript ToughMix Low ROX (Quanta BioScience; https://www.quantabio.com/), 0.2 µL 100 µM of the primer Zika 1086 (5′- CCGCTGCCCAACACAAG –3′), 0.2 µL 100 µM of the primer Zika 1162 (5′-CCACTAAYGTTCTTTTGCAGACAT-3′), 0.04 uL 25 uM Zika Probe, and 6.56 µL Millipore water. The Zika Probe used was 5Cy5/AGCCTACCT/TAO/TGACAAGCAGTCAGACACTCAA/3IAbRQSp/, where TAO is a proprietary internal quencher and the Iowa black (3IAbRQSp) is also a quencher. The reactions consisted in a first step at 50°C for 3 min, followed by a second step at 95°C for 10 min, and then 40 cycles of 5 s at 95 °C and 45 s at 60 °C.

References

1. Lanciotti RS, Kosoy OL, Laven JJ, Velez JO, Lambert AJ, Johnson AJ, et al. Genetic and serologic properties of Zika virus associated with an epidemic, Yap State, Micronesia, 2007. Emerg Infect Dis. 2008;14:1232–9. 10.3201/eid1408.080287 PubMed http://dx.doi.org/10.3201/eid1408.080287
